# *Drosophila* Insulin-Producing Cells Are Differentially Modulated by Serotonin and Octopamine Receptors and Affect Social Behavior

**DOI:** 10.1371/journal.pone.0099732

**Published:** 2014-06-12

**Authors:** Jiangnan Luo, Oleh V. Lushchak, Philip Goergen, Michael J. Williams, Dick R. Nässel

**Affiliations:** 1 Department of Zoology, Stockholm University, Stockholm, Sweden; 2 Department of Neuroscience, Uppsala University, Uppsala, Sweden; Lancaster University, United Kingdom

## Abstract

A set of 14 insulin-producing cells (IPCs) in the *Drosophila* brain produces three insulin-like peptides (DILP2, 3 and 5). Activity in IPCs and release of DILPs is nutrient dependent and controlled by multiple factors such as fat body-derived proteins, neurotransmitters, and neuropeptides. Two monoamine receptors, the octopamine receptor OAMB and the serotonin receptor 5-HT_1A_, are expressed by the IPCs. These receptors may act antagonistically on adenylate cyclase. Here we investigate the action of the two receptors on activity in and output from the IPCs. Knockdown of OAMB by targeted RNAi led to elevated *Dilp3* transcript levels in the brain, whereas 5-HT_1A_ knockdown resulted in increases of *Dilp2* and *5*. OAMB-RNAi in IPCs leads to extended survival of starved flies and increased food intake, whereas 5-HT_1A_-RNAi produces the opposite phenotypes. However, knockdown of either OAMB or 5-HT_1A_ in IPCs both lead to increased resistance to oxidative stress. In assays of carbohydrate levels we found that 5-HT_1A_ knockdown in IPCs resulted in elevated hemolymph glucose, body glycogen and body trehalose levels, while no effects were seen after OAMB knockdown. We also found that manipulations of the two receptors in IPCs affected male aggressive behavior in different ways and 5-HT_1A_-RNAi reduced courtship latency. Our observations suggest that activation of 5-HT_1A_ and OAMB signaling in IPCs generates differential effects on *Dilp* transcription, fly physiology, metabolism and social interactions. However the findings do not support an antagonistic action of the two monoamines and their receptors in this particular system.

## Introduction

Insulin and insulin-like growth factors (IGFs) are evolutionary conserved peptides that regulate development, growth and aspects of physiology in a broad range of animals [1]–[9]. In *Drosophila*, eight insulin-like peptides (DILP1–8) have been identified as likely ligands of a single insulin tyrosine kinase receptor [5], [6], [8], [10], [11]. In adult *Drosophila* the different DILPs, and thus insulin/IGF signaling (IIS), are of vital importance in the regulation of reproduction, metabolic homeostasis, resistance to stress and life span [11]–[15]. Additionally, attraction to food odors and feeding behavior are modulated by DILPs [16]–[18]. A cluster of 14 insulin-producing cells (IPCs) in the pars intercerebralis of the brain express DILP2, 3 and 5, which are secreted into the circulation via axon terminations in the corpora cardiaca, anterior aorta, foregut and anterior midgut as well as the crop [11], [12], [19].

In adult flies the activity in IPCs and thus production and release of DILPs is under control by fat body-derived diffusible molecules such as DILP6 and the leptin-like cytokine Unpaired 2 (Upd2) [20], [21]. Systemic release of these factors from the fat body is nutrient-dependent. Hence, when the fly feeds the increased levels of circulating carbohydrate and amino acids are sensed by adipocytes in the fat body, which induces signaling to the IPCs. In addition several neurotransmitters such as GABA and serotonin, as well as the neuropeptides corazonin, short neuropeptide F and *Drosophila* tachykinin [22]–[27] act on the brain IPCs. Except for the inhibitory transmitter GABA it is, however, not known what triggers the signaling by these substances to the IPCs. A portion of the GABAergic system in the pars intercerebralis seems to be inactivated by circulating Upd2 after feeding and thereby tonic inhibition of the IPCs is lifted (via the action of Jak/Stat signaling) which facilitates DILP release [20].

Another neurotransmitter implicated in the regulation of IPC activity in *Drosophila* is the biogenic amine octopamine [28]. Activation of an octopamine receptor, OAMB (OAMB-K3 splice form), in IPCs was found to promote sleep in *Drosophila* by stimulating adenylate cyclase and production of cyclic AMP (cAMP) [28], [29]. However, there is no evidence that sleep modulation is caused by release of DILPs from the IPCs. In fact, a later paper showed that insulin signaling has no effect on the sleep/wake state, whereas increased octopamine signaling to IPCs lead to increased circulating triglyceride levels which is DILP dependent [30]. Thus, octopamine and OAMB seem to play a role in activating IPCs, and this activation produces responses in sleep and metabolism, but only the latter is insulin-dependent. Here we decided to further investigate the role of OAMB in IPC activation and subsequent insulin signaling using metabolism and behavior as readouts.

Previously we demonstrated a role of one of the serotonin receptors, 5-HT_1A_, in regulation of *Drosophila* IPCs [22]. This receptor commonly inhibits adenylate cyclase (AC), and thus decreases levels of cyclic AMP (cAMP) and thereby diminishes activity of protein kinase A (PKA) (See reviews [31]–[33]). The OAMB receptor (K3 splice form) can both increase intracellular Ca^2+^ and activate adenylate cyclase and thus elevate cAMP and activate PKA [28], [34], [35]. The possible convergence of the two monoamine receptors on adenylate cyclase signal transduction lead us to compare the action of OAMB and 5-HT_1A_ on IPCs. Do the two receptors mediate antagonistic activity in IPCs via opposite actions on adenylate cyclase or do they act on independent intracellular systems?

To test this we employed the Gal4-UAS system [36] to direct OAMB and 5-HT_1A_-RNAi to IPCs and analyzed the effect on transcript levels of *Dilp2*, *3* and *5* and on carbohydrate metabolism and stress responses. We found that manipulations of the two receptors had differential effects on *Dilp* transcription, and mostly also in the other assays. Since both serotonin and octopamine are known to regulate social behavior in flies [37]–[42] we furthermore investigated the role of IPCs on aggressive and courtship behaviors by manipulating OAMB and 5-HT_1A_ in IPCs.

Our results do not support that octopamine and serotonin act antagonistically on the IPCs but suggest that activation of OAMB and 5-HT_1A_ in these cells induce differential effects on Dilp transcription, metabolism, stress resistance as well as male-male and male-female interactions.

## Results

### Processes from octopaminergic neurons superimpose IPC branches

**Figure 1:**
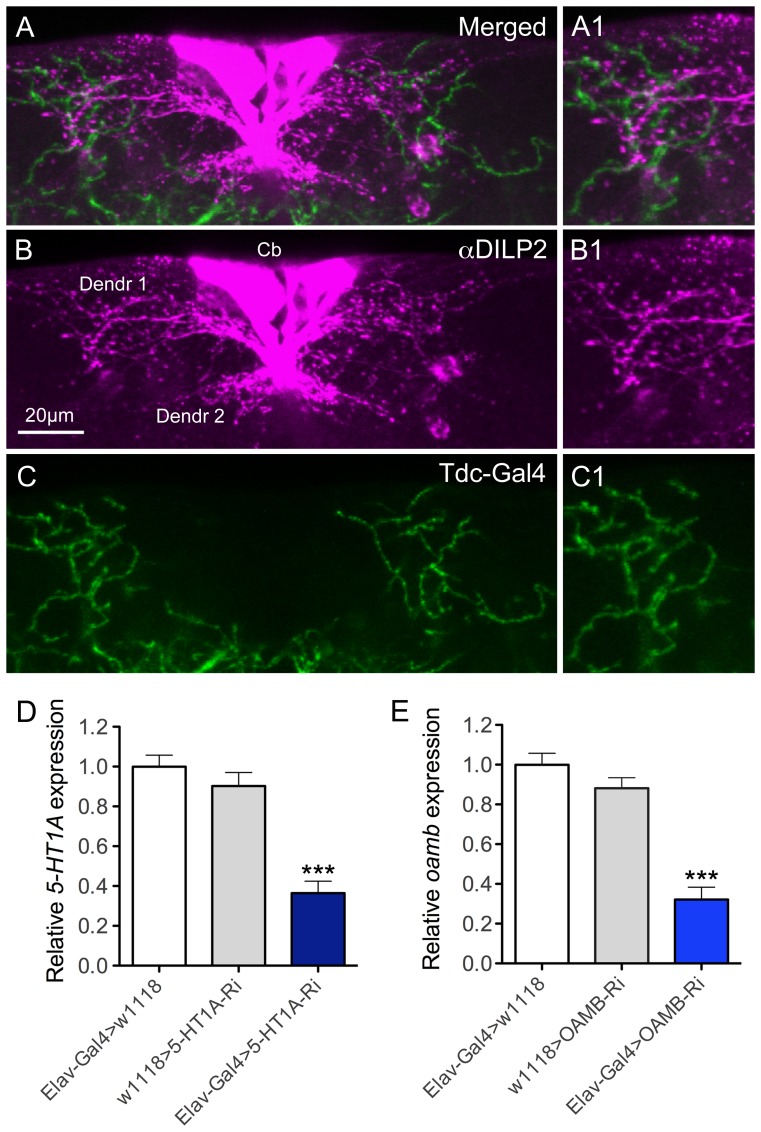
Branches of octopaminergic neurons superimpose those of insulin producing cells (IPCs). **A**–**C** Dissected adult brains were stained with rabbit anti-DILP2 (magenta) and octopaminergic neurons were visualized by *Tdc2*-Gal4 driving GFP (green). Cb, cell bodies of IPCs; Dendr1 and dendr2, presumed dendrites of IPCs. Scale bar = 20 µm. **A1–C1** Magnification of regions of interest in A–C. **D** and **E** The efficiencies of the UAS-5-HT_1A_- and UAS-OAMB-RNAi (UAS-OAMB-RNAi GD) were determined by using the pan-neuronal Elav-Gal4 driver and monitoring extracted RNA by qPCR. The transcript value for Elav-Gal4>w^1118^ was set as 1 in both assays. A significant knockdown (about 60–70% reduction) of 5-HT_1A_ and OAMB transcript was seen (***p<0.001 to both parental controls, One-way ANOVA, n = 2 samples for each genotype).

In a recent study it was shown that the IPCs express the OAMB-K3 receptor splice form, as determined by RT-PCR on RNA extracted from single neurons, and that a small set of octopamine-producing neurons, designated ASM, send axon processes to the IPCs [28]. The ASMs are a subpopulation of the *Tdc2*-Gal4 expressing neurons [28]. However, the octopamine distribution in relation to the presumed dendrites of IPCs was not revealed in detail. With application of DILP2 antiserum to brains with octopaminergic and tyraminergic neurons marked by *Tdc2*-Gal4 driven GFP, we found that some GFP-labeled branches superimpose those of the IPC dendrites in the pars intercerebralis of the brain (Fig 1). In the following we rely on published data [28] that the octopaminergic ASM neurons mediate activation of IPCs. Expression of the 5-HT_1A_ receptor in IPCs, and the possible innervation of IPCs by serotonergic neurons were shown previously [22].

### Knockdown of 5HT_1A_ or OAMB receptors in IPCs does not change cell size

To study the roles of the 5HT_1A_ and OAMB receptors in IPCs, we targeted RNAi for the two receptors to these cells using the Gal4-UAS technique [36]. First we determined the efficiency of the UAS*-5-HT_1A_-*RNAi (referred to as *5HT1A-*RNAi) and the UAS*-OAMB*-RNAi GD lines (referred to as *OAMB*-RNAi), by crossing these flies to the pan-neuronal *elav-*Gal4 driver, and performing qPCR to measure the level of *5-HT1A* and *OAMB* transcript (Fig. 1D, E). These flies were raised at 18°C until they eclosed, at which point the newly eclosed flies were collected and kept at 29°C for 5-7 days to obtain maximal expression from the Gal4-UAS system [36]. Compared with *elav-*Gal4 heterozygous controls (SEM±0.05), *5HT1A*-RNAi flies had 0.36-fold (SEM±0.06, P<0.005) of the normal *5-HT1A* RNA expression levels, while *OAMB*-RNAi flies had 0.32-fold (SEM±0.06, P<0.005) of the normal *OAMB* RNA levels (Fig. 1D, E).

We next used a *Dilp2*-Gal4 driver to specifically target receptor RNAi to the IPCs, using the same two RNAi lines. Since manipulations of IPC activity can lead to alterations of cell growth [43] we determined the effects of receptor-RNAi on the size of cell bodies of IPCs. No changes in cell size were observed after knockdown or either receptor (Fig. S1A,B). Therefore, we can exclude the possibility that physiological or behavioral phenotypes observed in subsequent experiments are due to gross developmental effects on cell morphology.

### Knockdown of 5-HT_1A_ or OAMB affects Dilp transcription in IPCs

**Figure 2:**
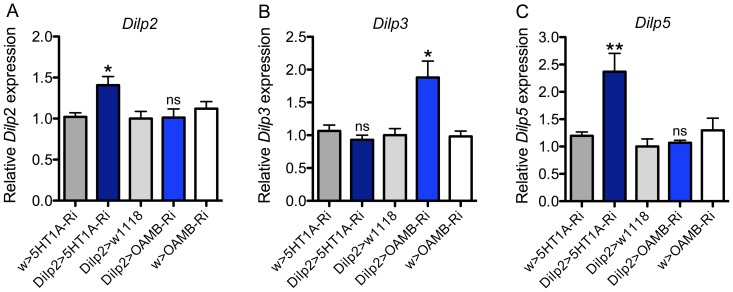
*Dilp* transcript levels in the brain are affected by 5-HT_1A_ and OAMB knockdown in IPCs. **A**–**C** Relative expression of *Dilp2, 3* and *5* transcripts after *5-HT_1A_*-RNAi and *OAMB*-RNAi (UAS-OAMB-RNAi GD) in IPCs was measured from head extracts of fed flies. The value for w^1118^>5-HT_1A_-RNAi was set to 1. The *Dilp2* and *5* transcripts increase significantly after 5-HT_1A_ knockdown, and *Dilp3* transcript increases after OAMB knockdown (one way ANOVA; **p<0.01, *p<0.05, ns, not significant; n = 3 samples for each genotype).

To test for effects of 5-HT_1A_ and OAMB knockdown on IPCs function we utilized qPCR to analyze *Dilp2, 3* and *5* brain transcript levels after targeted receptor RNAi in IPCs. Knockdown of 5-HT_1A_ in IPCs resulted in a significant increase in brain *Dilp2* and *Dilp5*, but not *Dilp3* transcript levels (Fig. 2). This is partly consistent with our earlier data where DILP2 immunoreactivity increased after 5-HT_1A_ knockdown both in fed and starved flies. On the other hand, knockdown of OAMB in IPCs (using *OAMB*-RNAi GD line) caused a significant increase in *Dilp3*, but not *Dilp2* and *Dilp5* (Fig. 2). These data indicate that each receptor would upon activation reduce specific *Dilp* transcription. However, it is important to note that the targeted RNAi does not mimic inactivation of the IPCs (or the two receptors), but induces a partial downregulation of receptor expression levels, which probably renders the IPCs slightly less responsive to octopamine or serotonin. The differential actions of serotonin and octopamine on IPCs in *Dilp* transcription might explain the different effects of OAMB and 5-HT_1A_ knockdown in some assays described in the following sections. It should be noted that we have no data to show effects on DILP release and therefore no clear readouts for alterations of IIS. Furthermore, these findings do not suggest clear-cut antagonistic actions of the two receptors since no decrease in *Dilp* RNA was detected for either receptor.

### Knockdown of OAMB in IPCs causes increased resistance to starvation

**Figure 3:**
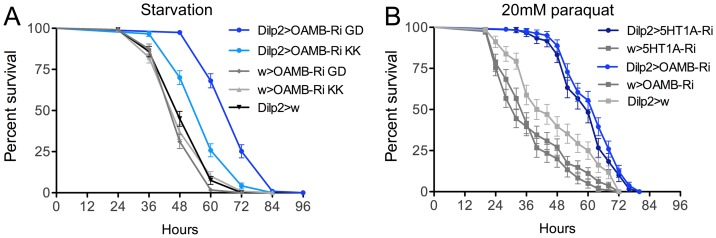
Stress responses after knockdown of octopamine receptor OAMB and serotonin receptor 5-HT_1A_ in IPCs. **A** Reduction of octopamine receptor OAMB level in IPCs increases resistance to starvation (seen by extended survival). OAMB was diminished in IPCs by driving two different OAMB-RNAi lines with *Dilp2*-Gal4. The Dilp2-Gal4>OAMB-RNAi GD flies showed about 50% percent increase of median lifespan compared with controls (***p<0.001 to both parental controls; Log rank test, Mantel-Cox; n = 120 for each genotype). We used the OAMB-RNAi GD lines hereafter for all other experiments. In an earlier study it was shown that 5-HT_1A_ knockdown decreased starvation resistance [22]. **B** Reduction of octopamine receptor OAMB or serotonin receptor 5-HT_1A_ levels in IPCs increases resistance to oxidative stress induced by feeding male flies 20mM paraquat in standard food. Both receptor knockdowns result in flies that display significantly increased survival under paraquat exposure (***p<0.001 to both parental controls, Log rank test, Mantel-Cox; n = 60–90 for each genotype).

Since previous evidence (see [22]) suggests that modulation of activity in IPCs results in changes in stress tolerance, we measured the starvation resistance in flies with diminished IPC OAMB receptor levels. Compared to controls, the flies with reduced OAMB expression displayed a significant increase in survival when exposed to starvation (Fig. 3A). Two independent UAS-OAMB-RNAi fly lines were tested in this assay and the same phenotypes were obtained. One of these RNAi lines (UAS-OAMB-RNAi GD), used in all experiments described above, produced a more prominent effect when crossed with the *Dilp2*-Gal4, and was therefore used also in subsequent experiments. Previously similar experiments were performed after 5-HT_1A_ RNAi in IPCs and resulted in decreased starvation resistance [22]. Thus OAMB and 5-HT_1A_ knockdowns in IPCs produce opposite effects on starvation resistance.

### Knockdown of OAMB or 5-HT_1A_ in IPCs both cause increased resistance to oxidative stress

It was shown that insulin signaling plays a role in resistance to oxidative stress; flies with ablated IPCs are more stress resistant [14]. We found that flies with diminished OAMB in IPCs were more resistant to oxidative stress induced by feeding 20mM paraquat in standard food (Fig. 3B). Also flies where 5-HT_1A_ was knocked down in IPCs displayed increased resistance to oxidative stress (Fig. 3B). Based on earlier findings [14] these effects on oxidative stress resistance suggest that both OAMB and 5-HT_1A_ activation in IPCs stimulate insulin signaling, or that the stress resistance phenotype is caused by other mechanisms mediated by the IPCs.

### Knockdown of OAMB or 5-HT1A in IPCs results in opposite effects on food intake

**Figure 4:**
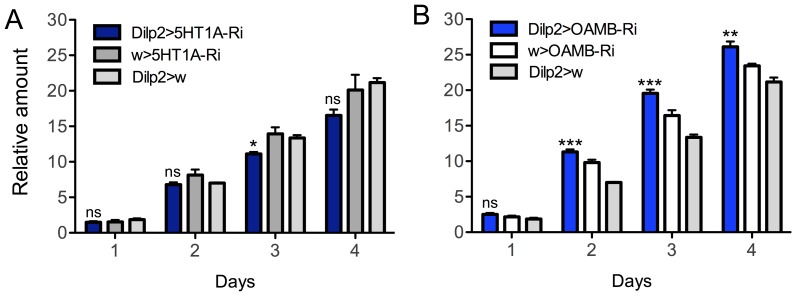
Knockdown of OAMB or 5-HT_1A_ in IPCs resulted in opposite phenotypes in a capillary feeding assay (CAFE). **A** Diminishing 5-HT_1A_ in IPCs (*Dilp2*-Gal4>*5-HT_1A_*-RNAi) significantly decreases food intake only during day 2. The units in the Y-axis are mm food consumed from the calibrated capillary tube (total volume 5 µL; see methods). The graph shows cumulative intake of food over 4 days (*p<0.05, n = 5-10 flies for each genotype in 3 replicates, One-way ANOVA). **B** Reduction of OAMB level in IPCs (*Dilp2*-Gal4>*OAMB*-RNAi) significantly increases food intake from the second day onwards (**p<0.01, ***p<0.001, n = 5–10 flies for each genotype in 3 replicates, One-way ANOVA).

Insulin signaling is known to modulate food search and feeding behavior in flies [16]–[18], [44], [45]. We determined whether food intake is affected by diminishment of OAMB or 5-HT_1A_ in IPCs by testing flies in a capillary feeding (CAFE) assay over 96 h. After 5-HT_1A_-RNAi a slight, but significant, decrease in food consumption was observed the third day (Fig. 4A). On the other hand OAMB knockdown resulted in increased food intake days 2–4 (Fig. 4B).

### Knockdown of 5-HT1A or OAMB in IPCs affect circulating and stored carbohydrate levels

Previous studies have shown that DILPs produced by the brain IPCs are important in the regulation of carbohydrate and lipid homeostasis [12], [14], [46]. Therefore we assayed carbohydrate levels to determine whether interference with OAMB and 5-HT_1A_ in IPCs affects systemic insulin signaling and metabolism.

**Figure 5:**
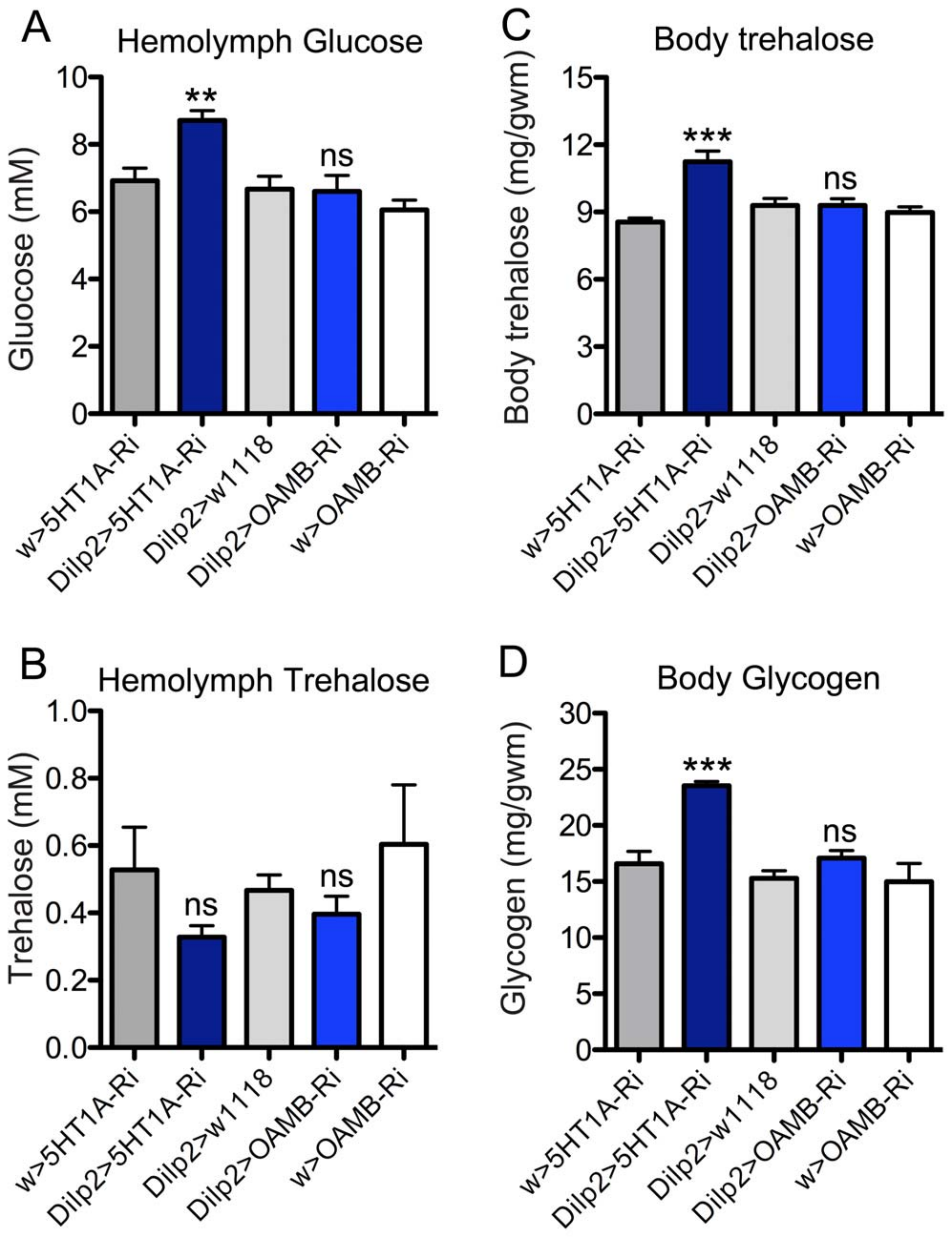
Levels of carbohydrates were affected by knockdown of 5-HT_1A_ in IPCs while no changes were observed for OAMB knockdown. A Hemolymph glucose levels in normally fed flies were higher after *5-HT_1A_*-RNAi in the IPCs (*Dilp2*>*5HT_1A_*-Ri) than controls, while no changes were observed for OAMB knockdown (*Dilp2*>*OAMB*-Ri). Data were analyzed with one way ANOVA, **p<0.01, n = 60-90 for each genotype, and experiments were performed in 6 independent replicates. B No significant changes of hemolymph trehalose levels were observed after *5HT_1A_*-RNAi or *OAMB*-RNAi in the IPCs. One-way ANOVA, ns, not significant; n = 60-90 for each genotype, and experiments were performed in 6 independent replicates. C Body trehalose levels in normally fed flies are higher after *5HT_1A_*-RNAi in the IPCs (*Dilp2*>*5HT_1A_*-Ri) than controls, but were not significantly changed after *OAMB*-RNAi in the same cells (*Dilp2*>*OAMB*-Ri). One way ANOVA, ***p<0.001, n = 60-90 for each genotype, and experiments were performed in 6 independent replicates. D Glycogen levels (whole body) in normally fed flies are higher after *5HT_1A_*-RNAi in the IPCs (*Dilp2*>*5HT_1A_*-Ri) than controls, but were not significantly changed after *OAMB*-RNAi in the same cells. One way ANOVA, ***p<0.001, n = 60–90 for each genotype, and experiments were performed in 6 independent replicates.

We measured the hemolymph levels of glucose and trehalose in *ad lib* fed male flies of different genotypes. Flies with 5-HT_1A_ knocked down in the IPCs displayed significantly increased hemolymph glucose levels, but not trehalose compared to parental controls (Fig. 5A, B). Knocking down OAMB did not influence circulating glucose or trehalose levels (Fig. 5A, B). Next we measured whole body trehalose and glycogen in the same experimental flies (normally fed) and found that 5-HT_1A_ knockdown in IPCs increase both stored carbohydrates, but OAMB-RNAi had no effect (Fig. 5C, D). Thus OAMB knockdown in IPCs does not seem to affect carbohydrate homeostasis in fed flies.

### Knockdown of 5HT_1A_ or OAMB in IPCs has no effect on body weight

Insulin signaling was shown to regulate larval development and growth in *Drosophila* [11], [12]. We therefore tested whether knocking down 5-HT_1A_ or OAMB in IPCs affected the body weight as measured in adult male flies. No effect was seen on body weight (Fig. S1C), similar to effects of GABA_B_ receptor knockdown in IPCs [27]. For the serotonin receptor this could be explained by the finding that 5-HT_1A_ appears to be absent in larval IPCs when growth occurs [22]. It is not known when OAMB starts to be expressed in IPCs.

### *5-HT1A* and *OAMB* regulate social behavior

It is known that serotonin and octopamine regulate aggression and mating behaviors in *Drosophila* [37]–[41], [47]. It was furthermore suggested that octopamine could modulate aggressive behavior via neurons releasing the cholecystokinin-like peptide drosulfakinin (DSK) [40]. One set of neurons that express DSK are the brain IPCs [45]. Thus, we tested he effects of *5-HT_1A_* and *OAMB* knockdown in IPCs on social behavior in male flies.

Aggression analysis experiments were executed by placing pairs of 5–7 day old males, raised in isolation, in a behavioral assay chamber, containing 1% agarose, and their interactions were monitored over a 20 min period. The total number of interactions for each fly was recorded, whether it involved aggressive or courtship behavior. The assayed male-male interactions consisted of eight distinct behaviors. Aggressive interactions were scored as either low or high-intensity engagements. Low intensity fighting (LIF) was scored as side-by-side pushing with a leg (side-fencing), face-to-face pushing with one leg (fencing), or quick wing flicking (wing flick); high intensity fighting (HIF) was graded as lunging (lunging), boxing face-to-face with the two front legs (boxing), as well as holding the wings up at a 30–45° angle (wing threat). Courtship behavior was marked as one-wing extended at a 90° angle (singing), circling to the posterior (circling), or bending the abdomen towards the other fly (abdomen bending).

**Figure 6:**
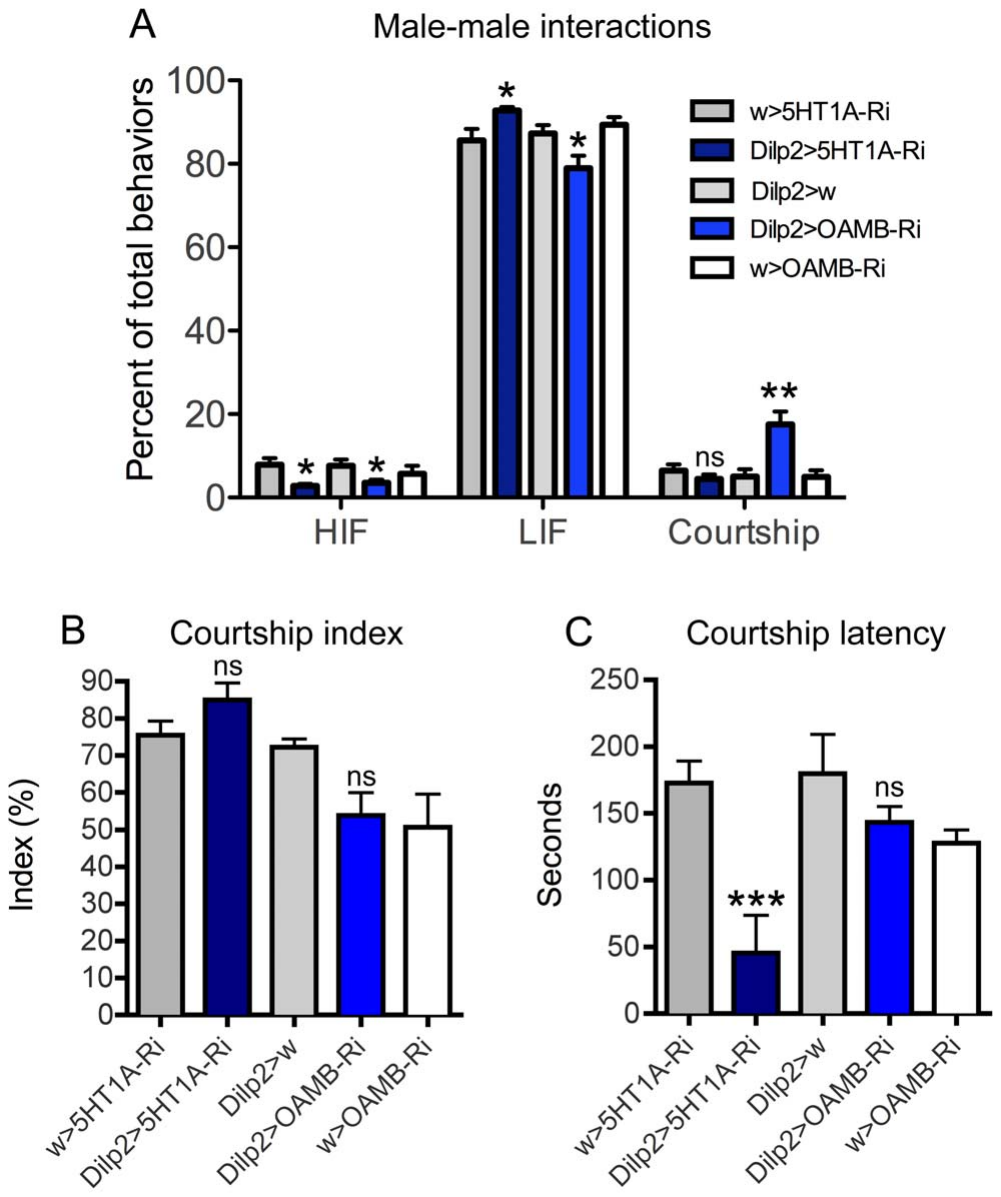
Social behaviors are affected by manipulations of 5-HT_1A_ and OAMB. **A** Aggressive interactions in controls, *5-HT1A*-RNAi and *OAMB*-RNAi knockdown males were determined. All males were between 5–7 days old. The types of behaviors were distributed into three categories, high intensity fighting (HIF), low intensity fighting (LIF) and courtship behavior (Courtship) and the percentage of each type is represented. In all instances the assay was repeated at least 10 times. (n = 20 males/treatment; * P<0.05, ** P<0.005 compared with controls, two-way ANOVA with Bonferroni post hoc test for multiple comparisons). **B** The percentage of time a male spent actively courting a 3–4 day old wild-type virgin female was determined for control, *5-HT1A*-RNAi and *OAMB*-RNAi knockdown males. **C** The amount of time, in seconds, before the first courtship behavior, or latency, was determined for control, *5-HT1A*-RNAi and *OAMB*-RNAi knockdown males introduced to 3–4 day old wild-type virgin females. In B and C n = 10 males per genotype; * P<0.05 ** P<0.005 compared with controls (one-way ANOVA with Bonferroni post hoc test for multiple comparisons).

For the HIF behaviors there was significant difference between controls, *5-HT_1A_* and *OAMB* knockdown males (P<0.05). Unlike controls, *OAMB-RNAi* males did not perform any wing threats (Table S1), while *5HT1A-RNAi* males performed significantly fewer lunging behaviors than controls (Table S1). Interestingly, when LIF behaviors were compared, *5HT1A-RNAi* males performed significantly more wing flicks and side fencing over a 20 min fighting bout than control males (Table S1). While the percentage of LIF behaviors performed by *OAMB-RNAi* males was significantly lower than controls (Fig. 6A), this was most likely due to the significant increase observed in courtship behaviors, since the actual number of LIF behaviors was not significantly different from controls (Table S1). Finally, knocking down *OAMB* had a significant effect on all scored mating behaviors, especially singing (Fig. 6A and Table S1).

Next we determined whether *5-HT1A* and *OAMB* also regulate male behavior towards virgin females. To test this, males were paired with wild-type virgin females and two aspects of male-female courtship were measured: latency and courtship index (see Materials and Methods). No effect was observed on courtship index for either receptor knockdown (Fig. 6B). When *5-HT1A* was knocked down there was a substantial decrease in latency (45.4 seconds, SEM±28.4, P<0.005) compared to either *Dilp2-GAL4* ^+/−^ (179.9 seconds, SEM±29.4) or *5-HT1A^RNAi^* ^+/−^ (172.8 seconds, SEM±16.4) controls (Fig. 6C). No significant effect was observed when *OAMB* was knocked down.

## Discussion

Our study shows that octopamine and serotonin differentially modulate the activity of brain IPCs in *Drosophila* via the receptors OAMB and 5-HT_1A_. The physiological readout of diminishing expression of either receptor in IPCs is complex and does not suggest convergence of the two receptors on the same downstream signaling cascade and also indicates involvement of IPC outputs additional to systemic IIS. Also the effects of targeted receptor-knockdown on social behavior may suggest that the two receptors act on independent pathways. We first discuss the effects of diminishment of OAMB and 5-HT_1A_ on physiology that may be regulated by IIS.

We find that knockdown of OAMB in IPCs affects some aspects of physiology that may be diagnostic of decreased systemic IIS, such as increased starvation and oxidative stress resistance and increased food intake (see Table 1). Surprisingly, however, OAMB-RNAi induced increased *Dilp3* transcription (and no effects on *Dilp2* and *5*). These findings would suggest that the activation of OAMB in IPCs in normally fed flies decreases DILP3 production, but stimulates some aspects of systemic IIS or produces phenotypes reminiscent of increased IIS. We did not detect any effect of OAMB knockdown on carbohydrate metabolism. We did not test triacylglyceride (TAG) levels here, but using a crude technique [22] to estimate of lipid levels in flies with OAMB knocked down in IPCs, we recorded a significant lipid decrease both in fed flies and flies starved for 24 h (not shown). A previous study found that OAMB mutants display decreased TAG levels, and activation of octopamine producing neurons increases TAG [30]. These authors also propose that the effect on TAG levels is via the IPCs since activation of octopamine neurons in a *Dilp2, 3* mutant background leads to less increase in TAG. In our earlier study we found that 5-HT_1A_ knockdown in IPCs results in decreased levels of stored lipid in fed and starved flies; the same was found in 5-HT_1A_ mutant flies [22]. Although the lipid assays performed in the two studies were different, it seems that knockdown of both OAMB and 5-HT_1A_ lead to decreased lipid levels. Possibly this suggests that activation of these receptors triggers increases stored lipids.

**Table 1:** Effects of OAMB and 5-HT_1A_ knockdown in various assays.

Assays with adult flies	Effect		Likely effect on insulin signaling^1^ (output)		Different effects
	OAMB-Ri	5-HT_1A_-Ri	OAMB-Ri	5-HT_1A_-Ri	
Starvation resistance	Increase	Decrease	Decrease	Increase	Yes*
Food intake (CAFE)	Increase	Decrease	Increase	Decrease	Yes*
Glucose (in circulation)	NE	Increase	NE	Decrease	Yes
Trehalose (in circulation)	NE	NE	NE	NE	No
Glycogen (stored)	NE	Increase	NE	Decrease	Yes
Trehalose (stored)	NE	Increase	NE	Decrease	Yes
*Dilp2* transcript	NE	Up	NE	Increase	Yes
*Dilp3* transcript	Up	NE	Increase	NE	Yes
*Dilp5* transcript	NE	Up	NE	Increase	Yes
Oxidative stress resistance	Increase	Increase	Decrease	Decrease	No
IPC cell body size	NE	NE	NE	NE	No
Body weight	NE	NE	NE	NE	No

NOTES: ^1^ This assumes that targeted receptor RNAi is similar to inactivating the receptor, which of course is not entirely correct. NE, no effect; * indicates opposite effect for the two knockdowns.

Although the role of OAMB in regulation of IIS remains somewhat unclear, our present data from manipulations of the 5-HT_1A_ in IPCs, combined with those of an earlier report [22], are more consistent with effects on systemic IIS. We show that 5-HT_1A_ knockdown increases *Dilp2* and *5* transcripts, reduces resistance to starvation, increases oxidative stress resistance, diminishes food intake and elevates levels of circulating and stored carbohydrates (Table 1). Taken together these findings indicate that targeted 5-HT_1A_ knockdown increases systemic IIS, and thus the activation of this receptor should decrease IIS. However, as seen in Table 1 our data from physiological readouts may suggest more complex effects of the receptor manipulations in IPCs.

One question raised in the present study was whether octopamine and serotonin act antagonistically on a shared downstream signal cascade to regulate IPC function. This was prompted by the possibility that both OAMB and 5-HT_1A_ act on adenylate cyclase, cAMP and PKA [28], [31]–[35]. We therefore compared the effects of receptor RNAi in several assays. In three of these we saw distinct effects of knocking down the two receptors: starvation resistance, food intake and transcription of *Dilp2, 3* and *5* (Table 1). In assays of carbohydrates only 5-HT_1A_ knockdown increased levels and no effect was seen with OAMB. Actually only in two assays, food intake and starvation resistance, did we observe opposite phenotypes after diminishing the two receptors. None of the receptor knockdowns diminished *Dilp* transcript levels, instead they increased RNAs of mutually exclusive *Dilps* (Table 1). Thus it is not possible to assign clear antagonistic effects of the two receptors in IPCs. One dilemma is that for each receptor we obtained incomplete or conflicting results. The effects of 5-HT_1A_-RNAi on metabolism suggest decreased IIS, which indicates that activation of the receptor should stimulate DILP release and increase IIS. However, the starvation resistance assay produced a phenotype opposite to the expected one after diminishment of 5-HT_1A_ [22]. In this context it should be noted that starvation resistance in the experimental flies could be caused by factors other than diminished IIS, such as increased locomotion or altered feeding [48], [49].

In the CAFE assay we noted opposite effects on food intake for the different receptor knockdowns. The effects of IIS on feeding are not clearly established in *Drosophila*. Some experiments suggest that silencing of the IPCs diminishes appetite and food intake when food has low calorie content [44], [48]. We showed in a recent study that silencing IPCs by expression of a constitutively active hyperpolarizing K-channel (Ork) increased intake of caffeine-spiked or sugar-free food [45], and conversely conditional activation of the IPCs with a temperature inducible TrpA1 channel diminishes food intake (J. Luo, Y. Liu and Nässel, in prep). In our present experiments 5-HT_1A_ knockdown in IPCs decreased food intake slightly. Thus, our data might indicate that serotonin receptor knockdown increases IIS and thereby diminishes feeding, and OAMB deficiency causes the opposite phenotype.

The effects on social behavior of OAMB and 5-HT_1A_ knockdown in IPCs are partly differential. Among the male interactions high intensity fighting is reduced by both knockdowns, low intensity fighting is up-regulated after 5-HT_1A_-RNAi and male-male courtship is up-regulated by OAMB-RNAi. In male-female courtship we only recorded a decrease in courtship latency for 5-HT_1A_-RNAi. There is no direct evidence that IIS affects aggression or courtship behaviors, although sexual receptivity and sexual attraction in female flies was shown to involve IPCs and IIS, respectively [50], [51], and aggression may depend on activity in neurons of the pars intercerebralis [52]. However octopamine was shown to regulate aggression in *Drosophila* via the cholecystokinin-like peptide drosulfakinin (DSK) [40]. DSK was detected in a subpopulation of the larval and adult IPCs [45], [53]. Thus, it is possible that the effects of manipulating OAMB in IPCs are caused by changes in DSK signaling rather than IIS, both in social behavior and physiology.

The action of OAMB and 5-HT_1A_ on IPCs may be complicated by the fact that OAMB-K3 is known to also activate intracellular Ca^2+^ in *Drosophila* [34], [35]. Thus, OAMB activation may cause more complex responses in IPCs including both Ca^2+^ and adenylate cyclase. Another factor that induces additional complexity is the role of OAMB in IPCs in regulation of sleep/wake activity, independent of IIS [30]. Also serotonin is implicated in sleep [54], [55] and the possible role of IPCs in mediating this action has not been investigated. Thus, if the IPCs play roles in regulatory activities other than systemic IIS, we might expect that phenotypes obtained after manipulating the two monoamine receptors are complex.

There are several important questions for the future. What are the triggers of octopamine and serotonin action on IPCs and how are the IPCs integrated into octopaminergic and serotonergic modulatory pathways? Octopamine has been extensively investigated in insects and crustaceans and is known to act as a neuromodulator and neurohormone with pleotropic functions, including roles in modulation of muscle and neurons, appetite, ovulation, aggression, sleep, and learning and memory [27], [34], [39], [56], [57], [58]. Octopamine is also involved in modulation of flight and escape jumping in *Drosophila* [56],[59]. Since octopamine seems central in modulation of energy demanding activities a role in regulation of IPCs and IIS is not surprising. Serotonin is also known to play multiple roles in *Drosophila* physiology and behavior: visual and olfactory learning [60], [61], courtship and mating [38], [62], aggression [37], [47], sleep and circadian activity [54], [55], olfaction [63] and feeding [64]. It is therefore important to design experiments to reveal which octopaminergic and serotonergic pathways that act on the IPCs and under what conditions they do so.

In summary, we established differential effects of OAMB and 5-HT_1A_ actions on IPCs in flies that suggest that octopamine and serotonin play distinct roles in modulation of these important neurosecretory cells. However, our data indicate that each receptor may trigger complex activity in the IPCs, some of which seem not to affect IIS.

## Materials and Methods

### Fly strains and husbandry

All flies were reared at 25°C on standard yeast, corn meal agar medium [according to Bloomington *Drosophila* Stock Center (BDSC), Bloomington, IN] under 12∶12 h light:dark conditions. Flies with other original genetic backgrounds were backcrossed into w^1118^ background for four generations before experiments, and w^1118^ flies were used as controls in crosses in all experiments. Fly stocks for behavior experiments were maintained on Jazz-mix *Drosophila* food (Fisher Scientific) containing sucrose, corn meal, 10% yeast, agar, benzoic acid, methyl paraben and propionic acid, and maintained at 25°C, 60% humidity, on a 12∶12 light:dark cycle. To delay Gal4 expression flies crossed to a Gal4 driver were kept at 18°C, and once the progeny eclosed they were shifted to 29°C for at least 5–7 days before any behavior assays were performed.

The following Gal4 lines were used: *Tdc2*-Gal4 [65] (J. Hirsh, Charlottesville, VA), *Dilp2*-Gal4 [12] (E. Rulifson, Stanford, CA) and *elav*-Gal4 (BDSC). We used the UAS lines: UAS-*OAMB*-RNAi (two different lines with stock ID 2861 and 106511) and UAS-*5-HT1A*-RNAi [stock ID 106094; see [22]] from the Vienna *Drosophila* RNAi Center (VDRC), Vienna, Austria, and UAS-*mcd8-gfp* are from BDSC. For mating behavior females of a *CSORC* strain was used. This is a lab wild type strain created by crossing Canton-S and *Oregon-R* wild type strains (BDSC).

### Antisera and immunocytochemistry

For immunocytochemistry adult *Drosophila* heads were dissected in 0.1 M sodium phosphate buffer (PB), pH 7.4 and fixed in ice-cold 4% paraformaldehyde in 0.1 M PB for 2-4 h and dissected adult brains were used for whole mount immunocytochemistry.

Incubation with primary antiserum for whole mount tissues was performed for 48 h at 4°C. The following primary antisera were used: rabbit antiserum to *Drosophila* insulin-like peptide 2 (anti-DILP2) at 1∶4000 (gift from M. Brown, Athens, GA), rabbit and mouse anti-GFP (Invitrogen) were used at 1∶1000. For detection of primary antisera Cy3-tagged goat anti-rabbit antiserum (Jackson Immuno Research) and Alexa-488 tagged goat anti-mouse (Invitrogen) were used at 1∶1000.

### Body weight

For each genotype at least three groups of 10 male flies were weighed on a Mettler MT5 Microbalance (Mettler Toledo, Switzerland) to obtain wet weight, and the average weight was calculated.

### Stress assays

Male and female flies (4–6 d old) were collected for starvation experiments. Flies were placed individually in 2 ml glass vials with 500 µl of 0.5% aqueous agarose and dead flies were monitored every 12 h. These starvation experiments were run in three replicates with at least 30 flies of each genotype per replicate.

For the oxidative stress assay, 20–30 flies were placed into vials with 5 ml of standard food containing 20 mM paraquat (methyl viologen, Sigma, St Louis). Dead flies were recorded every 4 h or 12 h. These oxidative stress assays were run in two replicates. In all the above experiments survival curves and statistics (Log rank test; Mantel-Cox) were made using Prism GraphPad 5.0.

### Assays of carbohydrates

Male flies (4–6 days old) were used to measure concentrations of circulating glucose and trehalose together with stored glycogen and whole body trehalose. Pre-weighed flies were decapitated and hemolymph was collected by centrifugation (3000 g, 6 min). Hemolymph was used to measure circulating glucose and trehalose whereas whole bodies were used for determination of glycogen and stored trehalose. All parameters were measured with a glucose assay kit involving glucose oxidase and peroxidase (Liquick Cor-Glucose diagnostic kit, Cormay, Poland). Trehalose was converted to glucose by porcine kidney trehalase (Sigma T8778) and glycogen by amyloglucosidase from *Aspergillus niger* (Sigma 10115). Glucose and trehalose are expressed as concentration in hemolymph whereas glycogen and body trehalose are given as amount per wet weight. All genotypes were tested in 3 independent replicates (15–20 flies of each genotype in each sample) and one-way ANOVA was used to compare differences between genotypes.

### Capillary feeding (CAFE) assay

The capillary feeding (CAFE) assay was conducted according to Ja and others [66] with slight changes. Male flies were placed into 1.5 ml Eppendorf tubes with an inserted 5 µl capillary tube with 5% sucrose, 2% yeast extract and 0.1% propionic acid. Three food-filled capillaries were inserted as controls in identical tubes without flies. The final consumption of food was determined as the diminished food level (in mm) minus the average diminishment in control capillaries (due to evaporation). Daily food consumption was measured every 24 h and calculated cumulatively over 4 consecutive days. These experiments were run in three replicates with 10 flies of each genotype for each replicate.

### Quantitative real-time PCR (qPCR)

Relative amounts of *Dilp* 2, 3 and 5 RNA in heads of male flies were measured by qPCR. RNA was isolated from 4 biological replicate samples of each genotype tested. One µg of total RNA was used for cDNA synthesis. cDNA was synthesized in triplicates, which were subsequently pooled and diluted for qPCR. Expression of genes of interests was measured relative to that of the housekeeping gene *Actin88* (*Act*) using an ABI Prism 7000 instrument (Applied Biosystems) and a SensiFAST SYBR Hi-ROX Kit (Bioline) under conditions recommended by manufacturer. Each analytical and standard reaction was performed in three technical replicates. The levels of *Dilp2*, *3* and *5* and *Act* were measured with the following primer pairs (all 5′ to 3′):

Dilp2F: AGCAAGCCTTTGTCCTTCATCTC and

Dilp2R: ACACCATACTCAGCACCTCGTTG;

Dilp3F: TGTGTGTATGGCTTCAACGCAATG and

Dilp3R: CACTCAACAGTCTTTCCA-GCAGGG;

Dilp5F: GAGGCACCTTGGGCCTATTC and

Dilp5R: CATGTGGTGAGATTCG-GAGC;

Act88F: AGGGTGTGATGGTGGGTATG and

Act88R: CTTCTCCATGTCGTCCCAGT.

For analysis of the efficiency of the two RNAi lines by qPCR we used a slightly different protocol. Relative expression levels of three housekeeping genes (*EF-1, Rp49* & *RpL11*) and of the genes of interest were determined with qPCR. Each reaction, with a total volume of 20 µl, contained 20 mM Tris/HCl pH 9.0, 50 mM KCl, 4 mM MgCl_2_, 0.2 mM dNTP, DMSO (1∶20) and SYBR Green (1∶50000). All qPCR experiments were performed in duplicates; for each primer pair a negative control with water and a positive control with 5 ng/µl of genomic DNA was included on each plate. Analysis of qPCR data was performed using MyIQ 1.0 software (Bio-Rad) as previously reported [67]. Differences in gene expression between groups were analyzed with ANOVA followed by Fisher's PLSD test where appropriate. P<0.05 was used as the criterion of statistical significance. The following primers were used to amplify reference housekeeping genes:

EF-1F: 5′-GCGTGGGTTTGTGATCAGTT-3′,

EF-1R: 5′-GATCTTCTCCTTGCCCATCC-3′;

Rp49F: CACACCAAATCTTACAAAATGTGTGA-3′,

Rp49R: 5′-AATCCGGCCTTGCACATG-3′;

RpL11F: 5′-CCATCGGTATCTATGGTCTGGA-3′,

RpL11R: 5′-CATCGTATTTCTGCTGGAACCA-3′.

To amplify 5-HT1A the primers were as follows:

F: 5′-GTGGCCAATACC-3′, R: 5′-ATCTGGTTGCCAGAAGTGCT-3′.

To amplify OAMB the primers were as follows:

F: 5′- TTGGCCGTCCTACCCTTCT-3′,

R: 5′-CGGTCCAGTGATATGGCACAC-3′.

### Image analysis

Specimens were imaged with Zeiss LSM 510 META and Zeiss LSM 780 confocal microscopes (Jena, Germany) using 20x, 40× oil or 63× oil immersion objectives. Confocal images were processed with Zeiss LSM software for either projection of z-stacks or single optical sections. Images were edited for contrast and brightness in Adobe Photoshop CS3 Extended version 10.0. For cell size determination, the outline of cell body was delineated manually and its area determined using Image J [from NIH, Bethesda, MD, USA (http://rsb.info.nih.gov/ij/)]. For each genotype neurons of 8–15 male flies from 3 independent crosses were measured.

### Aggression assay

Newly emerged male flies were collected and isolated for 5 to 7 days at 29°C, 60% humidity, on a 12∶12 light:dark cycle. Behavioral tests were carried out at room temperature with 60% humidity in cylindrical behavioral chambers (2 cm by 2.5 cm; height × diameter), filled with 1% agarose to 1.5 cm in height to maintain proper humidity. Two male flies were anesthetized using an ice-water bath before being transferred to a behavioral chamber. After a recovery period of at least 3 minutes, a camera (Panasonic HDC-SD90), positioned above the chamber, was used to record activity for a minimum of 30 minutes. After the 3 min recovery period the behavioral interactions between the males was scored for 20 minutes. Distinct stereotypic aggressive interactions were scored as described by Nilsen *et al.* [15] and Chen *et al.* [68]. Aggressive interactions were further scored as either low or high-intensity engagements. Low intensity fighting (LIF) was scored as side-by-side pushing with a leg (side-fencing), face-to-face pushing with one leg (fencing) or quick wing flicking (wing flick); high intensity fighting (HIF) was graded as frontal lunging (lunging) or boxing face-to-face with the two front legs (boxing), holding the wings at a 30° angle (wing threat), as well as chasing one another (chasing). Courtship behavior (CB) was marked as one-wing extended at a 90° angle (singing), circling to the posterior (circling), tapping the abdomen (tapping). At least 10 replicates were conducted for each genotype.

### Mating behavior assay

Newly eclosed males were collected and aged in isolation for 5 to 7 days, at 29°C, 60% humidity, on a 12∶12 light:dark cycle. Individual males and 3-4 day old virgin wild type *CSORC* females were then transferred to a behavioral chamber, using ice-water anesthetization. After a recovery period of at least 3 minutes, a camera (Panasonic HDC-SD90), positioned above the chamber, was used to record activity for a minimum of 30 minutes. After the 3 min recovery period the behavioral interactions between the males and females was scored for 20 minutes or until copulation occurred. Scoring of the courtship behaviors was performed as described by Becnel *et al*. [38]. Latency, courtship index as well as the frequency of mating behaviors were measured. Latency was calculated by counting the time it took a male to initiate mating and courtship index is calculated as the percentage of time a male spends actively courting a female over a 20 minute period (Seconds spent actively courting/(1200 seconds – Latency seconds). At least 10 replicates per genotype were conducted.

### Statistical Analysis

All statistical analyses were performed using GraphPad Prism 5.0. Survival data were analyzed by Log rank test with Mantel-Cox post test, for quantification of immunofluorescence, lipid values and body weights we used One-way ANOVA with Tukey's comparison or two way ANOVA depending on analysis (see Figure legends for details). Data are presented as means and standard error of means (SEM). For behavior analysis we used ANOVA with appropriate post hoc analysis for multiple comparisons.

## Supporting Information

Figure S1 Knockdown of 5HT_1A_ or OAMB in IPCs does not affect of cell sizes of IPCs, or body weight. **A** IPCs were visualized by anti-DILP2 labeling after 5-HT_1A_ and OAMB knockdown in IPCs (using *Dilp2*-Gal4). No difference in immunolabeling intensity or cell body size was noted. Scale bar 10 µm. **B** Quantification of cell body size of IPCs after 5-HT_1A_ and OAMB knockdown in IPCs. Adult male flies of 4-6 d age were used (ns, not significant; Student's T-test, n =  brains of 7-9 flies for each genotype). **C** Adult body weight after 5-HT_1A_ and OAMB knockdown in IPCs. Adult 4-6d old male flies were weighed as described in Materials and Methods. (ns, not significant; one-way ANOVA; n = 40 flies for each genotype).
(TIF)

Table S1 Effects of OAMB- and 5-HT_1A_-RNAi on social behavior. Experimental conditions and statistics are described in legend of Fig. 6.
(TIF)
