# Supplementary figures and images for: Drosophila Insulin-Producing Cells Are Differentially Modulated by Serotonin and Octopamine Receptors and Affect Social Behavior
Source: PLoS One. 2014 Jun 12;9(6):e99732. doi: 10.1371/journal.pone.0099732 (PMC4055686; doi:10.1371/journal.pone.0099732)

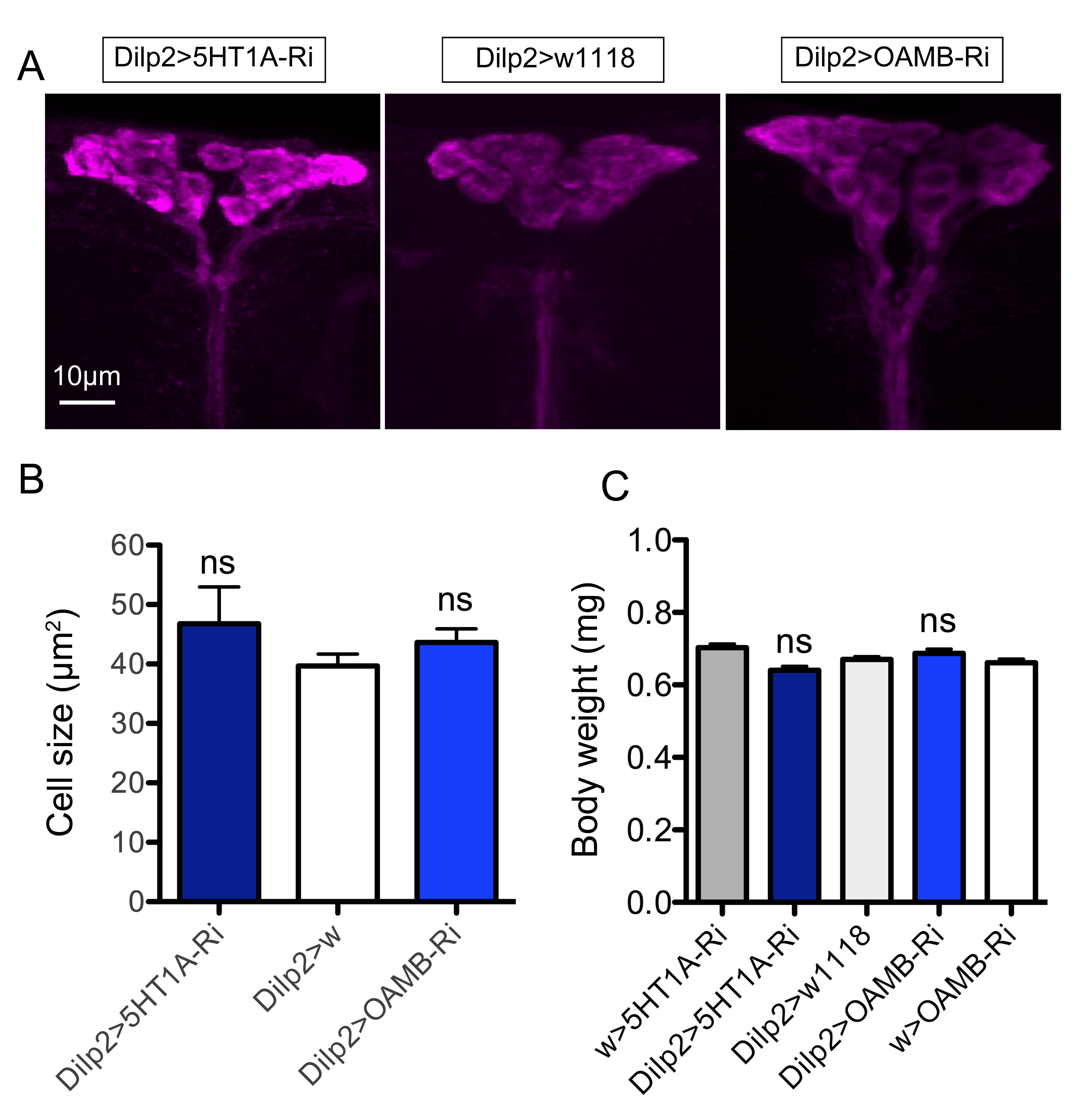

Supplement: Figure S1 — Knockdown of 5HT1A or OAMB in IPCs does not affect of cell sizes of IPCs, or body weight. A IPCs were visualized by anti-DILP2 labeling after 5-HT1A and OAMB knockdown in IPCs (using Dilp2-Gal4). No difference in immunolabeling intensity or cell body size was noted. Scale bar 10 µm. B Quantification of cell body size of IPCs after 5-HT1A and OAMB knockdown in IPCs. Adult male flies of 4-6 d age were used (ns, not significant; Student's T-test, n = brains of 7-9 flies for each genotype). C Adult body weight after 5-HT1A and OAMB knockdown in IPCs. Adult 4-6d old male flies were weighed as described in Materials and Methods. (ns, not significant; one-way ANOVA; n = 40 flies for each genotype). (TIF) [file pone.0099732.s001.tif]

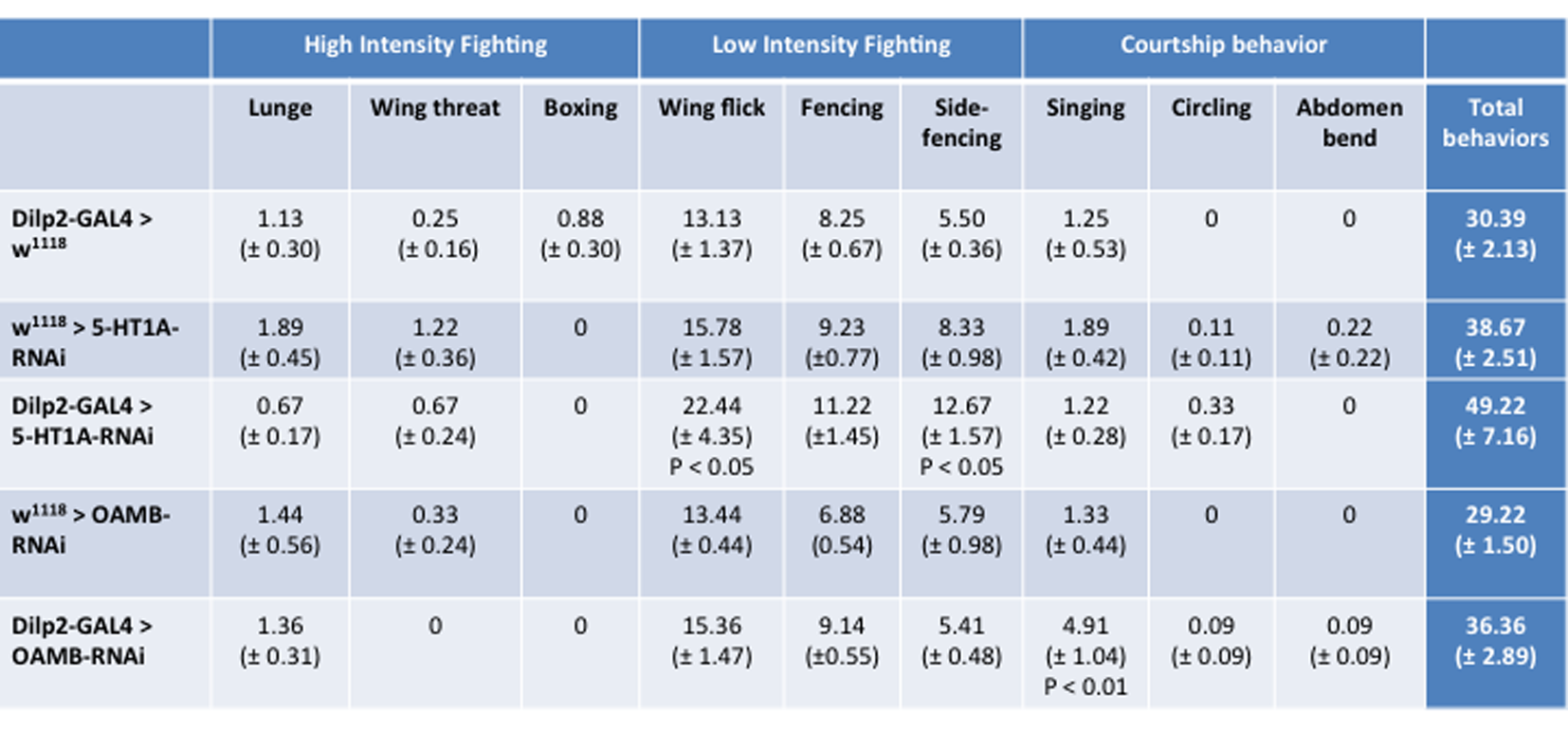

Supplement: Table S1 — Effects of OAMB- and 5-HT1A-RNAi on social behavior. Experimental conditions and statistics are described in legend of Fig. 6. (TIF) [file pone.0099732.s002.tif]
